# Physiological and molecular responses of a resistant and susceptible wheat cultivar to the fungal wheat pathogen *Zymoseptoria tritici*

**DOI:** 10.1371/journal.pone.0308116

**Published:** 2024-10-04

**Authors:** Amir Mirzadi Gohari, Fateme Ghiasi Noei, Amin Ebrahimi, Mohammad Amin Ghanbari, Fardad Didaran, Mohsen Farzaneh, Rahim Mehrabi

**Affiliations:** 1 Department of Plant Pathology, Faculty of Agricultural Sciences and Engineering, College of Agriculture and Natural Resources, University of Tehran, Karaj, Iran; 2 Agronomy and Plant Breeding Department, Faculty of Agriculture, Shahrood University of Technology, Semnan, Iran; 3 Department of Horticultural Science, School of Agriculture, Shiraz University, Shiraz, Iran; 4 Photosynthesis Laboratory, Department of Horticulture, Aburaihan Campus, University of Tehran, Tehran, Iran; 5 Medicinal Plants and Drugs Research Institute, Shahid Beheshti University, Tehran, Iran; 6 Department of Biotechnology, College of Agriculture, Isfahan University of Technology, Isfahan, Iran; National Taiwan University, TAIWAN

## Abstract

*Zymoseptoria tritici* is the causal agent of Septoria tritici blotch (STB), one of the most economically destructive wheat foliar diseases. In this study, we explore the physiological and molecular changes elicited in two wheat cultivars with divergent responses (Taichung 29 = susceptible, and Shafir = resistant) upon infection by *Z*. *tritici*. Our aim is to uncover novel insights into the intricate mechanisms that govern wheat defense against *Z*. *tritici* infection. Our quantitative histopathological study showed that H_2_O_2_ accumulated in the resistant cultivar to a higher degree compared to the susceptible cultivar at the biotrophic and switching phase. Additionally, we combined qPCR with a targeted quantitative HPLC technique to evaluate the expression profiles of 13 defense-related genes and profile the polyphenolic compounds induced differentially in the STB susceptible and resistant cultivar. Our finding indicated that five out of 13 genes were strongly up-regulated in the resistant cultivar compared with that of the susceptible one at eight days post-inoculation (dpi), corresponding to the transition phase present in the infection process of *Z*. *tritici*. Finally, our targeted HPLC analysis demonstrated that the traced phenolic compounds were highly elevated in the susceptible cultivar infected by *Z*. *tritici* compared with that of the resistant cultivar. In conclusion, our comprehensive analysis unveils a robust defense response in the resistant wheat cultivar Shafir, characterized by heightened H_2_O_2_ accumulation, significant up-regulation of key defense-related genes during the transition phase, and a distinct profile of polyphenolic compounds, shedding light on the intricate mechanisms contributing to its resistance against *Z*. *tritici*, thereby providing valuable insights for the development of more resilient wheat varieties.

## Introduction

*Zymoseptoria tritici* (Desm.) Quaedvlieg & Crous [1] causes Septoria tritici blotch (STB), which is a notorious disease of wheat, threatening global food security. Under favourable environmental conditions for disease development, STB leads to huge economic yield losses and results in decreasing grain quality [2]. Control of STB is based on a combination of chemical compounds and using resistant cultivars bearing *Stb* resistance genes [3]. Nevertheless, both employed measures pose some limitations in providing an efficient control. These limitations include emerging fungicide resistant isolates in natural populations [4], hindering disease management, and breaking the conferred resistance through *Stb* genes [5]. It is worth noting that *Z*. *tritici* has an active sexual cycle allowing this damaging pathogen to overcome unfavorable environmental (fungicide resistance) and biological (host resistance) conditions [6]. Consequently, it is of great importance to understand the molecular basis of the host-pathogen relationship and manipulate host resistance towards this harmful pathogen.

*Z*. *tritici* has a hemibiotrophic lifestyle with two distinct invasion phases comprising a stealth biotrophic phase followed by a necrotic phase. The fungus seems to remain endophytic during the biotrophic stage lasting for 7–10 days before a sudden switch to the advanced necrotrophic phase, coinciding with the occurrence of disease symptoms [7]. Nevertheless, recent studies indicated that asynchronous development of *Zymoseptoria tritici* infection in wheat exists, and an epiphytic growth of this fungus before the invasion of the host plant is demonstrated [8, 9]. The necrotrophy is suppressed in an incompatible interaction where fungal biomass remains unchanged, while it increases in a compatible interaction [7]. Therefore, it seems that the transition phase is a crucial determinant of compatibility or incompatibility in the *Z*. *tritici-*wheat interaction. This switch has features reminiscent of programmed cell death (PCD) corresponding with a loss of cell-membrane integrity, dramatic increases in apoplastic metabolites, and a sharp increase in fungal biomass in infected leaves [10]. However, Rudd et al. (2008) demonstrated that resistance in the *Z*. *tritici*-wheat pathosystem is not associated with PCD, an effective resistance mechanism toward biotrophic fungal pathogens such as powdery mildew (*Blumeria graminis*) or rust (*Puccinia graminis* f.sp. *tritici* [11–13]. Interestingly, no sign of programmed cell death, a hallmark of an incompatible interaction between biotrophic pathogens and cereal hosts, was observed during the biotrophic infection process of *Z*. *tritici*. It is worth mentioning that recent studies indicated that the prevention of penetration through stomatal closures is a crucial step in suppressing STB development, similar to switching from biotrophy to necrotrophy [14, 15].

The *Z*. *tritici*–wheat relationship follows the gene-for-gene model via the recognition of the effector AvrStb6, which is recognized by the corresponding resistance protein Stb6 [6, 16]. It is presumed that this recognition leads to a wide array of defensive responses in cv. Shafir, which harbors the resistance gene *Stb6*. These responses suppress further colonization of *Z*. *tritici* IPO323 beyond the transition phase [14].

Induced expression of defense-related genes is a common response to fungal infection and molecular markers can indicate compatibility/incompatibility [17]. Some studies have compared transcriptional reactions of cereal plants such as wheat and rice during compatible and incompatible interactions with hemibiotrophic plant pathogens [18, 19]. These investigations revealed that there is a differential expression of defense-related genes between susceptible and resistant plants [20]. Additionally, it is reported that differential changes in the timing of the activated defense-related genes depend on compatibility/incompatibility [21] and even types of the applied race/cultivar [22, 23].

Rapid accumulation of phenolic compounds at the attempted infection sites by the fungal pathogen is another tactic employed by plants to combat pathogen attack [24]. Plant phenolics are secondary metabolites constituting structurally diverse products generated through the shikimate—phenylpropanoid pathways. They are pre-existing barriers implicating in plant defense strategies. These compounds have potent antioxidant properties and roles in neutralizing the free radical molecules generated during the oxidative burst [25]. Several lines of evidence suggest that phenolic compounds are quantitatively and temporally different between incompatible and compatible interactions. It claimed that they could be applied as promising markers to determine susceptibility and resistance of pathosystems such as the *Z*. *tritici*-wheat interactions [14, 26–29].

Revealing the molecular basis of *Z*. *tritici*-wheat interactions may aid in defining the differences between compatibility and incompatibility. More importantly, discovering the molecular mechanisms underlying defense responses would facilitate the development of effective management strategies against STB. Therefore, the purpose of this research was to extend previous efforts to determine molecular markers associated with compatibility and incompatibility in *Z*. *tritici*-wheat pathosystem enabling us to delineate the differences between compatible and incompatible responses that occurred in wheat attacked by the *Z*. *tritici*. [22, 23, 30]. To accomplish this, we performed a quantitative histochemical analysis coupled with staining with DAB and tryptophan blue to monitor the occurred events in the resistant and susceptible plants. Additionally, we combined qPCR with a targeted quantitative HPLC technique to either evaluate the expression profiles of 13 defense-related genes in the investigated wheat cultivars upon infection by *Z*. *tritici* and to profile the polyphenolic compounds induced differentially. Genes for expression profiling were elected to cover the key defense-related genes, playing an essential role in host-microbe interactions, including pathogenesis-related protein PR-1 (*PR-1*), peroxidase (*Per*), phenylalanine ammonia-lyase (*PAL*), and serine carboxypeptidase (*SCP*) [22]; β-1,3-endoglucanase (*PR-2*), thaumatin-like protein (*PR-5*) and lipoxygenase (*LOX*) [30]; chitinase (*Chit*) [31]. Three antioxidant enzymes comprising catalase (*CAT*), mitochondrial manganese superoxide dismutase (*MnSOD*) and glutathione peroxidase (*GPX*) were selected [32]. Mitogen-activated protein kinases (*TaMPK3*), known to transcriptionally activate at the symptomless biotrophic stage in both compatible and incompatible interactions in *Z*. *tritici*-wheat pathosystem, were also included [12]. A protein disulphide isomerase (*PDI*), a molecular chaperone, was chosen since previous report showed that it was strongly induced in resistant cultivar challenged with the *Z*. *tritici* [30]. Time points were selected to represent consecutive stages of *Z*. *tritici* pathogenesis, comprising 2, 4 days post-inoculation (dpi) as well as 12, 16 and 21 dpi corresponded with biotrophy and necrotrophy, respectively. Eight dpi was considered as the transition phase expected to play a central role in determining the outcome of the interaction in this pathosystem. Additionally, the amount of three phenolic chemicals traced in this study, investigated recently in the AvrStb6-Stb6 interaction, was quantified in the applied resistant and susceptible plants [14].

## Materials and methods

### Fungal isolate

The fully sequenced *Z*. *tritici* reference strain IPO323 was used [33].This strain was stored at -80 °C and was re-cultured on potato dextrose agar (PDA) (Sigma-Aldrich Chemie, Steinheim, Germany) at 18 °C once desired for experimentation. Yeast-like spores were produced in yeast glucose broth (YGB) medium (yeast extract 10 g/L, glucose 30 g/L) after placement in a shaker incubator set at 125 rpm (Innova 4430; New Brunswick Scientific, Nijmegen, The Netherlands) at 18 °C and their concentrations were calculated by a disposable hemocytometer (VWR, The Netherlands).

### Plant material and infection assay

Wheat cultivar (cv.) Shafir expressing resistance against avirulent IPO323 due to the presence of resistance Stb6 protein interacting with AvrStb6 in IPO323 was utilized as a resistant plant while wheat cv. Taichung 29 was susceptible plant [34]. Plants used in this study were cultivated in a greenhouse under controlled conditions, maintained at a temperature of 18/16 °C (day/night rhythm) and a relative humidity (RH) of 70%. They were grown until reaching the stage where the first leaves were fully unfolded. For each pot, 3–5 seeds were initially sown, and after 7 days, three seedlings were selected in each pot, each with their first fully unfolded leaves. It’s important to note that inoculation was performed at this stage, prior to the emergence of the second set of leaves. The inoculum was produced in YGB (yeast extract 10 g/L, Glucose 30 g/L) at 18 °C for 7 days in a shaker incubator adjusted at 125 rpm (Innova 4430; New Brunswick Scientific, Nijmegen, The Netherlands), and yeast-like spores were obtained after centrifugation at 330 ×g and two washing steps to remove the residual medium. Subsequently, the spore concentrations were adjusted to 1 × 10^7^ spores mL^-1^ and the resulting suspension was supplemented with 0.15% Tween 20 as a surfactant (MERCK^®^, Nottingham, UK). In each biological replicate, seeds of cvs. Shafir and Taichung 29 were sown in a 5 cm pot until the first leaves were fully unfolded. Infection assays were performed by a hand sprayer till run off and inoculated plants were incubated in transparent plastic bags for 48 hours under 100% RH and then transferred to a greenhouse compartment (22 °C, relative humidity > 90%- and 16-hours light).

### Infection biology

The infection biology of *Z*. *tritici* was examined by collecting inoculated leaves at 2, 4, 8, 12, 16, and 21 dpi for compatible (IPO323-cv. Taichung 29) and incompatible interactions (IPO323-cv. Shafir). These leaves were then cleared using 100% ethanol, acetic acid, and glycerol (3:1:1) for 30 minutes at 80 °C to remove the leaves’ natural color (Shetty et al., 2003). In the next step, microscopic slides made from the infected area of the leaves were created using a lactophenol solution (Shetty et al., 2003). A light microscope from Olympus^®^ was used to examine the prepared slides (Olympus, Tokyo, Japan). Based on the previously described technique, a study of the quantitative developmental phases of the strains used *in planta* was performed [35]. These assays were carried out in three biological samples and was repeated three times independently.

### *In planta* detection of H_2_O_2_ and cell death

The 3,3-diaminobenzidine (DAB, D-8001, Sigma) staining technique was used to detect H_2_O_2_ *in planta* [36]. At 2, 4, 8, 12, 16, and 21 days post-inoculation, the infected leaves were collected and placed in a container with a dilute acid (pH 3.8) containing 1 mg/ml DAB while remaining in the dark. The container was kept in a desiccator equipped with a vacuum pump to generate a suction force of 0.2 bar for 30 min, and this was maintained overnight. The following day, a clearing technique was carried out to remove chlorophyll using 100% ethanol, acetic acid, and glycerol (3:1:1) for 30 min at 80 °C [35]. According to Koch and Slusarenko (1990), dead cells and fungal structures were stained by trypan blue [37]. In this assay, 3 infected leaves per each biological sample were collected. These tests were carried out in three biological samples and was repeated three times independently.

### Image processing

Using PlantCV, the integrated optical density (IOD) of DAB- or trypan-blue-stained leaf regions was determined [38]. For this, a multiclass naive Bayes procedure based on RGB pixel values was applied. Three staining strengths (low, medium, and high), background, and unstained areas were all covered in the classes. These categorizations are based on how strongly the color is present in the stained tissues; these strengths were first visually categorized, and then Photoshop was used to detect the color spectrum. The three intensities level were defined by machine learning using a table of red, green, and blue color values made up of 50 pixels in each class, with each column representing a different class [39]. Finally, using machine learning data, hundreds of pixels in all leaves were classified into these classifications. For each of the classes, probability density functions (PDFs) were created using the table. Adobe Photoshop was applied to extract the RGB values from the leaf photos (Version 23.1.0).

### Quantification of gene transcription

Transcript accumulation of 13 genes of interest (S1 Table) was investigated using quantitative reverse transcription PCR (qRT-PCR). Each replicate contained three separate seedlings that carried one fully unfolded leaf. Three infected leaves per each replicate were harvested at 2, 4, 8, 12, 16, and 21 dpi, flash frozen and ground in liquid nitrogen using a mortar and pestle. This assay was conducted in three biological samples (three different plants). Total RNA was extracted from ground leaves using the RNeasy plant mini kit (Qiagen, location, USA), and subsequently, DNA contamination was removed using the DNA-free kit (Ambion, Cambridgeshire, U.K.). First-strand cDNA was synthesized from two μg of total RNA primed with oligo (dT) using the Invitrogen SuperScript III Reverse Transcriptase according to the manufacturer’s instructions. One μl of the resulting cDNA (15 ng) was used in a 25 μl PCR reaction using a QuantiTect SYBR Green PCR Kit and run and analyzed using an ABI 7500 Real-Time PCR System. The relative expression of each gene was initially normalized with the constitutively expressed wheat *beta-tubulin* gene and then calculated based on the comparative C(t) method described previously [40]. The R package pheatmap was applied to draw the heatmaps, with the special parameters cluster rows = TRUE to classify the genes based on the hierarchical clustering (https://cran.r-project.org/web/packages/pheatmap/index.html).

### Extraction of phenolic compounds and targeted HPLC-UV analysis

The first infected leaves of cvs. Shafir and Taichung 29 were harvested at 2, 4, 8, 12, 16, and 21 dpi (days post-inoculation), flash-frozen, and ground in liquid nitrogen. For each biological sample, three infected leaves were collected. Subsequently, the powdered material from three infected first leaves of each pot was combined to create one biological replicate. This approach ensured that each replicate represented a homogenized mixture of leaves from the same pot, minimizing potential variability between replicates originating from different pots. Subsequently, 10 mL of methanol was added to each powdered leaf, incubated for 30 min in an ultrasonic bath, and centrifuged at 13000 ×g for 5 min. The extraction solvent was evaporated using a rotary evaporator at 45 °C, and then the dry participate was dissolved in 1 mL of methanol. This assay was conducted in three biological samples (three different plants). The HPLC profile analysis of methanolic extracts was done by Waters 2695 Alliance HPLC system equipped with 996 PDA detector to monitor phenolic compounds as previously reported [27]. The mobile phase included of (A), methanol+ 0.02%TFA; and (B), HPLC grade water+ 0.02% trifluoroacetic acid (TFA); with the flow-rate of 0.5 mL min^-1^, on the C18 column (Novapack C18, 4.6 × 15 mm, 4 μm) were used in the gradient program for 60 min. The Peaks were monitored at 200–400 nm wavelength and identification of each phenolic compound was carried out by comparison of its spectra and retention time with the 14 targeted commercial standards indicated in the S2 Table.

### Statistical analysis

This study employed a factorial design conducted in a completely randomized design with three replications to ensure robustness and reliability of the results. Statistical analyses were performed using SPSS 26 software (IBM SPSS, Armonk, NY, USA), and the effects of the independent variables were assessed through a two-way ANOVA. Mean differences were further examined using the LSD test at a significance level of 1% to compare the levels of the factors. Furthermore, meticulous melting and amplification curve analyses were conducted on the gene expression data to ensure data accuracy. Heatmap clustering analysis was performed using the heatmap.2 function in the gplots (Version: 3.1.3.1) R-package, employing the coefficient of Euclidean distance with the Ward method for clustering.

## Results

### Disease symptoms development

The selected time points correspond with consecutive stages of pathogenesis in the Z. *tritici* IPO323-wheat interaction covering from inoculation to the onset of pycnidial formation. No symptoms were observed at earlier time points (2 and 4 dpi) corresponding to the biotrophic stage in both interactions. Chlorosis lesions initiating from leaf tips formed on the leaves of cv. Taichung 29 (susceptible cultivar) at 8 dpi which is at the switch to necrotrophy while inoculated leaves of cv. Shafir (resistant cultivar) remained green at 8 dpi. Formed lesions on cv. Taichung 29 expanded over time and eventually developed into large necrotic lesions covered by asexual fruiting bodies containing abundant pycnidiospores (14–21 dpi) whereas small chlorotic flecks appeared on the leaves of cv. Shafir at 21 dpi. S1 Fig displays symptoms once the final samples were harvested on day 21 after inoculation.

### Infection biology

**Fig 1:**
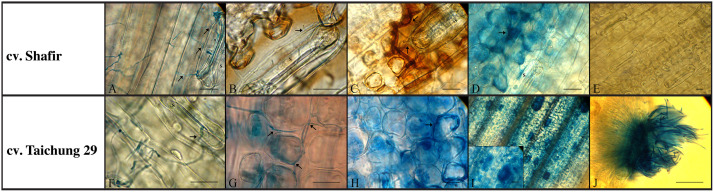
Histopathological development occurred in the cv. Shafir (incompatible interaction), and Taichung 29 (compatible interaction) infected by WT IPO323 strain. (A and F) The fungal strain germinated by 2 dpi and pierced directly through stomata (S stands for the stomata and arrow reflects the germinated spores). (B and G) Accumulation of H_2_O_2_ as observed by the formation of red-brown staining in the incompatible context (upper panel) and intercellular growth of the penetrated infective hyphae in the compatible interaction (lower panel), respectively at 4 dpi. (C and H) Extensive accumulation of H_2_O_2_ in the cv. Shafir infected by *Zymoseptoria tritici* IPO323, blocks further hyphal growth while intercellular hyphal growth of WT strain was seen in the cv. Taichung 29 at 8 dpi. (D) Cytoplasmic-like shrinkage happened in the incompatible context at 8 dpi. (I) Initiating the formation of the immature asexual flask-shaped fruiting bodies in the compatible interaction at 12 dpi. (E and J) Healthy cells were observed in the incompatible context while the release of asexual spores (pycnidiospore) was found in the compatible interaction at 21 dpi. The scale bars are 20 μm.

**Fig 2:**
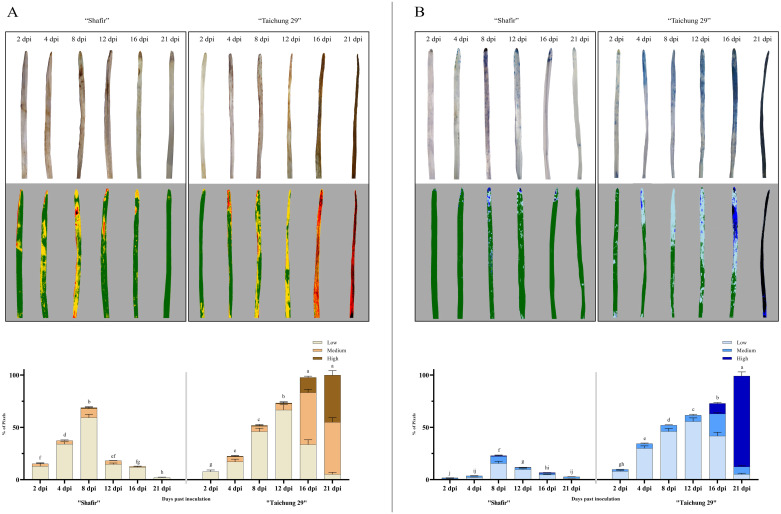
Accumulation of hydrogen peroxide (H_2_O_2_) following staining by DAB signified by the red-brown areas (A) and the occurrence of cell death indicated as dark-blue areas stained by the trypan blue (B). The wheat leaves of cvs. Shafir and Taichung 29 were inoculated by *Z*. *tritici* IPO323 strain by a hand sprayer. Pictures were taken at 2, 4, 8, 12, 16, and 21 dpi. The images were analyzed by the PlantCV software to obtain the integrated optical density (IOD), displaying the percentage of the stained cells by DAB and trypan blue. Data are indicated as mean ± SD (Standard Deviation) of three biological samples from three independent experiments.

Microscopical observation showed that the landed spores in both interactions germinated by producing thin germ tubes and penetrated via stomata by 2 dpi (Fig 1A and 1F). At investigated time points, the proportion of germinated spores was low (15–29%; S3 Table), but it did not differ substantially between the incompatible (cv.Shafir-IPO323) and compatible interactions (cv. Taichung 29-IPO323). Our observations led to the identification of three categories that depict the directions in which germ tubes produced by spores are developing. These include the germ tubes growing towards a stoma, germ tubes ending on a stoma, and germ tubes growing past a stoma. H_2_O_2_ accumulated in stained cells 2–8 dpi to a greater extent in the incompatible interaction compared with susceptible plants infected with *Z*. *tritici* IPO323 (Figs 1B and 1C; 2A). This event happened particularly around the substomatal cavity where the penetration occurred (Fig 1B). H_2_O_2_ gathered around the fungal hyphae, attempting to enter the apoplastic space, and no further hyphal growth was seen (Fig 1C). In the incompatible context at 8 dpi, cell collapse combined with cytoplasmic-like shrinking was also observed (Fig 1D). In the compatible interaction, intercellular hyphal growth was noticed at the 4–8 dpi, possibly as the generated H_2_O_2_ was insufficient to halt the hyphal progression (Fig 1G and 1H). The initial formation of the a-sexual fruiting body structures (Fig 1I) was seen 12 days post-inoculation, coinciding with tissue collapse and mature pycnidia releasing the yeast-like cells were observed at 21 dpi (Fig 1J).

### H_2_O_2_ accumulation and cell death occurrence

To investigate the functional significance of H_2_O_2_ and cell death in the resistant and susceptible plants, we evaluated the accumulation of H_2_O_2_ and the onset of cell death at several time intervals, including 2, 4, 8, 12, 16, and 21 dpi. In the incompatible interaction (cv. Shafir-IPO323), the temporal accumulation of H_2_O_2_ and cell death was discovered at low levels between 2–4 dpi, peaking at 8 dpi (Fig 2). Following the switch phase (8 dpi), low accumulation of H_2_O_2_ and low cell death was observed at the necrotrophic stages (12, 16, and 21 dpi). In contrast, susceptible plants (cv. Taichung 29) inoculated by the IPO323 (compatible interaction) displayed increasing H_2_O_2_ accumulation and cell death from 2 dpi onwards. The hallmarks of these plants were high levels of H_2_O_2_ accretions and the incidence of cell death at the necrotrophic development stages (12, 16, and 21 dpi). Therefore, high levels of H_2_O_2_ accumulations occur, which result in the infected leaves dying completely at 21 dpi (Fig 2A). The PlantCV tool was also employed to quantify the stained area of infected leaves by DAB. Our findings revealed that the incompatible interaction generates substantially more H_2_O_2_ than the susceptible plants during the transition stage (8 dpi) (Fig 2A; S4 Table). Similar results were obtained for trypan blue staining by applying the PlantCV program. At 8 dpi, cell death levels peaked in the incompatible interaction compared with the compatible interaction. As we expected, cv. Taichung 29 experienced high levels of cell death at the necrotrophic stages (onwards 8 dpi) (Fig 2B; S4 Table).

### Gene transcription profiling

The expression levels of genes involved in the defense response were assessed using the qRT-PCR technique in two wheat cultivars exhibiting contrasting responses to *Z*. *tritici* infection. Our analysis revealed significant differences in the relative expression of the studied genes between the two interactions, with the exception of PDI, which did not show statistical significance (Table 1; S5 Table).

**Table 1:** Reduced anova table indicating the interaction between wheat cvs. and *Zymoseptoria tritici*. The analysis derived from qRT-PCR data of gene expression of 13 wheat genes on two cultivar/*Zymoseptoria tritici* IPO323 combinations over six time points including 2, 4, 8, 12, 16, and 21 days post-inoculation covered the entire infection process of this damaging fungal pathogen.

Source of variance	DF	*CAT*	*Per*	*TaMPK3*	*PR*.*1*	*PR*.*2*	*PR*.*5*	*SCP*	*Chit*	*GPX*	*LOX*	*MnSOD*	*PAL*	*PDI*
Mean of square														
**Genotype**	1	8.4 *	2231 ***	76.89 ***	50**	3885***	229 ***	0.702***	313 ***	122.8 ***	8.213 ***	42.65 ***	0.205 ***	3.98 ^ns^
**Time course**	5	621***	2167 ***	70.20 ***	5819 ***	8327***	12182 ***	0.101 ***	5167 ***	894.0 ***	4.695 ***	50.25 ***	0.022 *	296.0 ***
† Genotype*Time	5	38.7**	1328 ***	13.73 ***	2582 ***	5669***	3431 ***	0.169 ***	3582 ***	9.4 ***	6.036 ***	5.15 ***	0.006 ^ns^	148.3 ***
**Error**	24	1.6	2.4	0.2	6	2	3	0.002	5	1.2	0.068	0.23	0.006	1.51

ns; non-significant.

*, ** and *** significantly at the probability level of 5%, 1%, and 0.1%, respectively.

^**†**^ Genotype*Time indicate cross interaction of Genotype and Time course.

The observed distinctions were highly significant, with a P-value < 0.001, except for CAT, which reached statistical significance at P < 0.05. These findings suggest a substantial modulation of the majority of the genes in response to *Z*. *tritici* attack. Furthermore, significant alterations in the expression levels of the tested genes were observed at the selected time points, with PAL showing significance at P < 0.05 and other genes at P < 0.001. This indicates that the transcript accumulation of the studied genes was statistically different in both interactions. Additionally, the interaction of genotype and time course for all examined genotypes, except for *PAL*, exhibited differential expression at a significance level of P < 0.01 (Table 1).

#### Genes expressed differentially during biotrophic and transition phases

**Fig 3:**
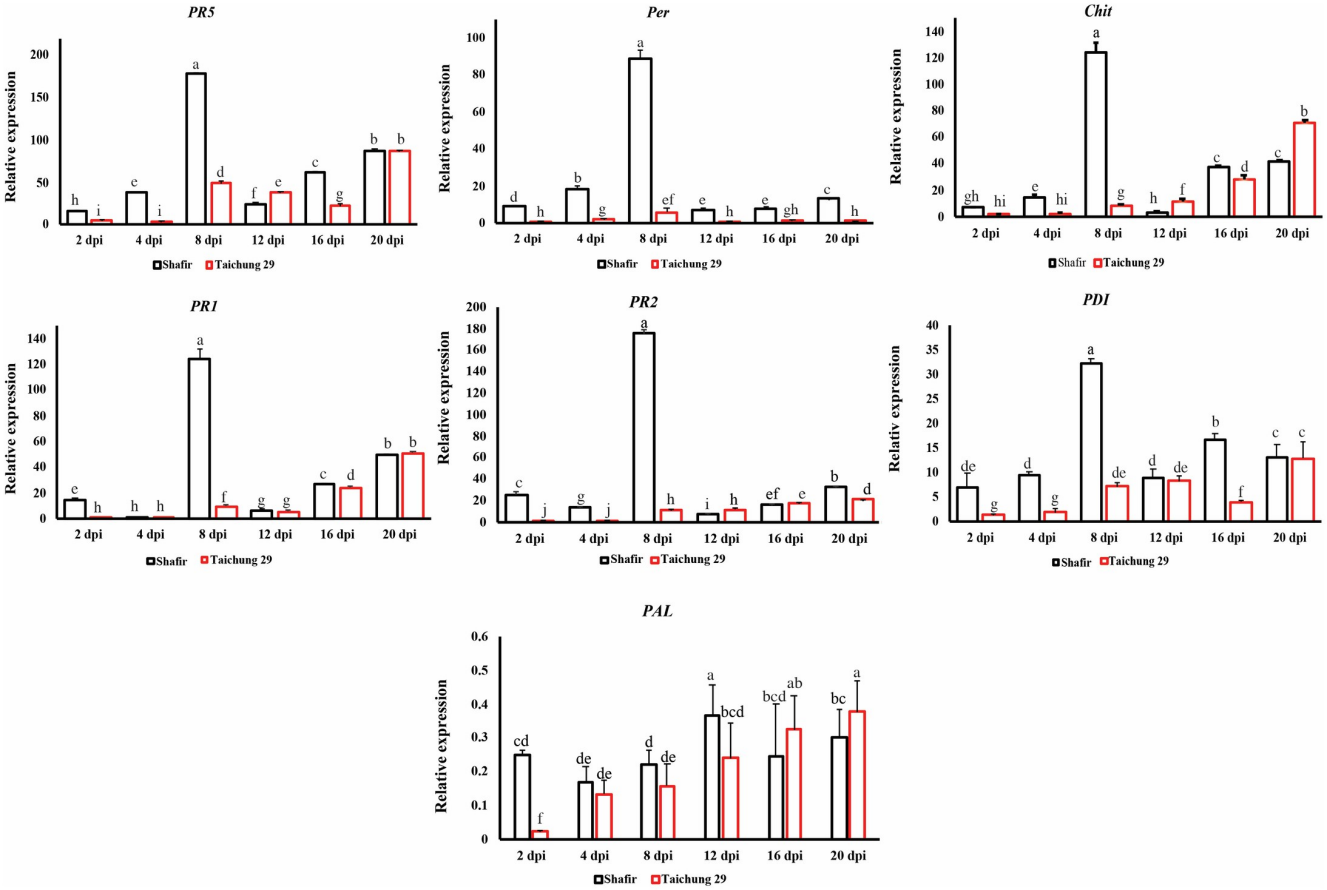
Relative *in planta* expression profiling of interesting wheat defense-related genes that are specifically up-regulated during the biotrophic and transition phases. Inoculated leaves of resistant cv. Shafir, as well as susceptible cv. Taichung 29 by *Zymoseptoria tritici* IPO323 was harvested at 2, 4, 8-, 12-, 16-, and 21-days post-inoculation. Subsequently, the expression level of targeted genes was initially normalized with the constitutively expressed wheat beta-tubulin gene and then calculated based on the comparative C(t) method.

The expression analysis demonstrated that seven defense-related genes, including *PR-1*, *PR-2*, and *PR-5*, *Per*, *Chit*, *PDI*, and *PAL* were significantly up-regulated in an incompatible interaction compared with that of the compatible interaction. *PR-5*, *Per*, and *Chit* follow the same expression patterns: gradually up-regulated at early time points (2 and 4 dpi), peaking at eight dpi, whereas *PR-1* and *PR-2* show the opposite trend: gradually down-regulated during biotrophy (2 and 4 dpi), followed by the highest peak at transition stage (8 dpi). There were no remarkable differences in gene expression of *PDI* during biotrophy in an incompatible interaction but was significantly higher than that of a compatible context. The highest transcript level recorded at 8 dpi was around 178, belonging to *PR-5* and *PR-2*, while the lowest transcript accumulation belonged to *PDI* (30). Lastly, *PAL* was expressed at a low level in both interactions ranging from 0.02–0.3. Nevertheless, this gene was significantly up-regulated at the incompatible interaction compared to the compatible one during the biotrophic stage (2 dpi) (Fig 3).

#### Genes have bimodal expression profiles

**Fig 4:**
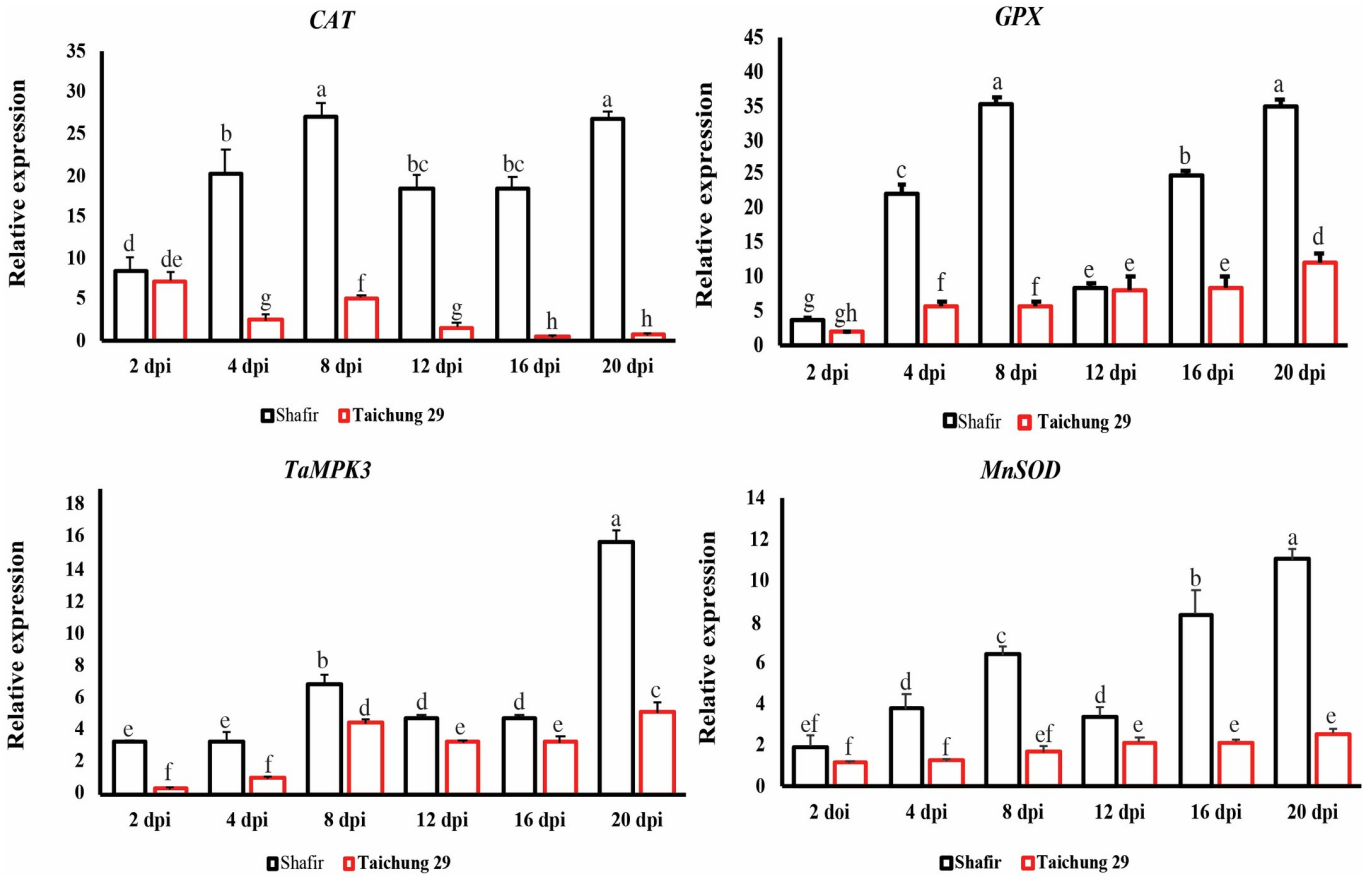
Relative *in* planta expression profiling of interesting wheat defense-related genes having bimodal expression pattern. Inoculated leaves of resistant cv. Shafir, as well as susceptible cv. Taichung 29 by *Zymoseptoria tritici* IPO323 was harvested at 2, 4, 8-, 12-, 16-, and 21-days post-inoculation. Subsequently, the expression level of targeted genes was initially normalized with the constitutively expressed wheat beta-tubulin gene and then calculated based on the comparative C(t) method.

In contrast to the previous genes, *CAT* and *GPX* showed a bimodal expression profile, indicating they were up-regulated steadily during the early stage of infection (2 and 4 dpi) in cv. Shafir, then dropped to 16 dpi. Notably, pycnidial formation, which typically occurs at 21 dpi in other cultivars such as Taichung, was not observed in the Shafir cultivar. Despite this, *CAT* and *GPX* were significantly up-regulated again at 21 dpi in Shafir, suggesting potential roles beyond pycnidial formation. *TaMPK3* and *MnSOD* exhibited the same trend, but the transcript accumulation of these genes at 21 dpi was significantly higher than that of the transition phase (8 dpi). The transcript accumulation of these genes varied from 6–16 in cv. Shafir challenged with IPO323 compared with that of cv. Taichung 29 (Fig 4).

#### Genes up-regulated in the compatible interaction

**Fig 5:**
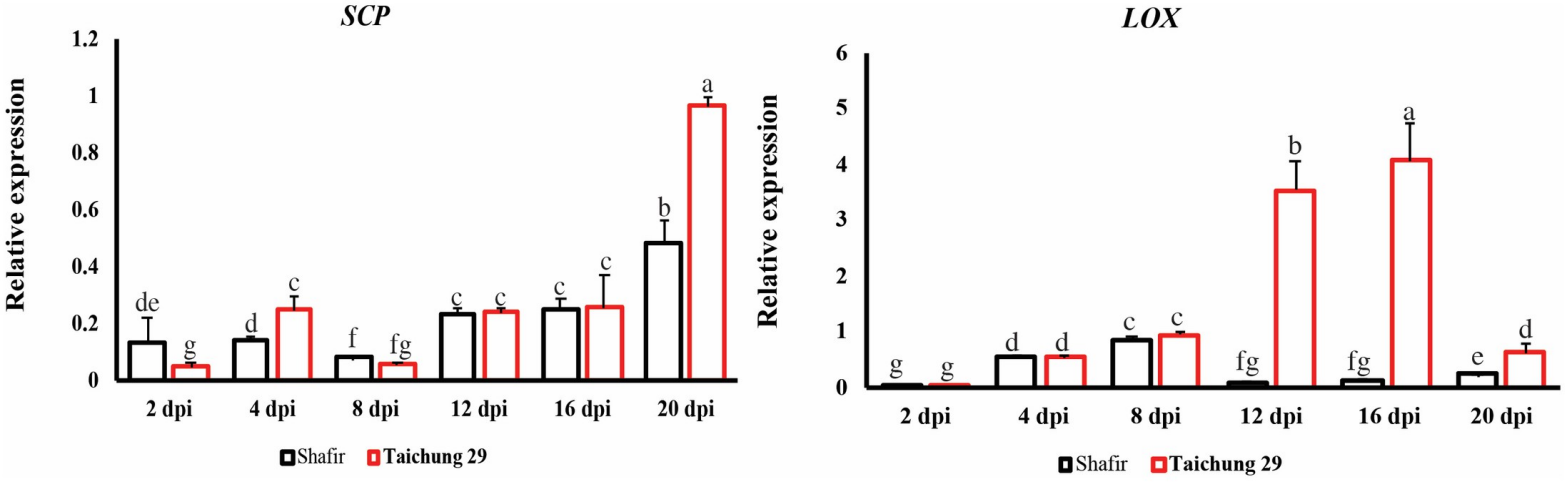
Relative *in* planta expression profiling of interesting wheat defense-related genes that are particularly up-regulated in the compatible interaction. Inoculated leaves of resistant cv. Shafir, as well as susceptible cv. Taichung 29 by *Zymoseptoria tritici* IPO323 was harvested at 2, 4, 8-, 12-, 16-, and 21-days post-inoculation. Subsequently, the expression level of targeted genes was initially normalized with the constitutively expressed wheat beta-tubulin gene and then calculated based on the comparative C(t) method.

Two genes, including *SCP*, and *LOX* were expressed at low levels *in planta*, but the highest peaks were found in the compatible interaction compared to the incompatible context. *SCP* was induced significantly at 2 dpi in cv. Shafir inoculated by IPO323 compared with that of cv. Taichung 29, but the highest transcript induction of this gene was noticed in the compatible interaction at the late stage of infection (21 dpi). There was no significant accumulation of *LOX* in both interactions during early time points by 12 dpi, where this gene was expressed greatly in cv. Taichung 29 challenged by IPO323 and continued to be expressed by 16 dpi, followed by a sharp reduction to 21 dpi (Fig 5).

#### Heat map analysis

**Fig 6:**
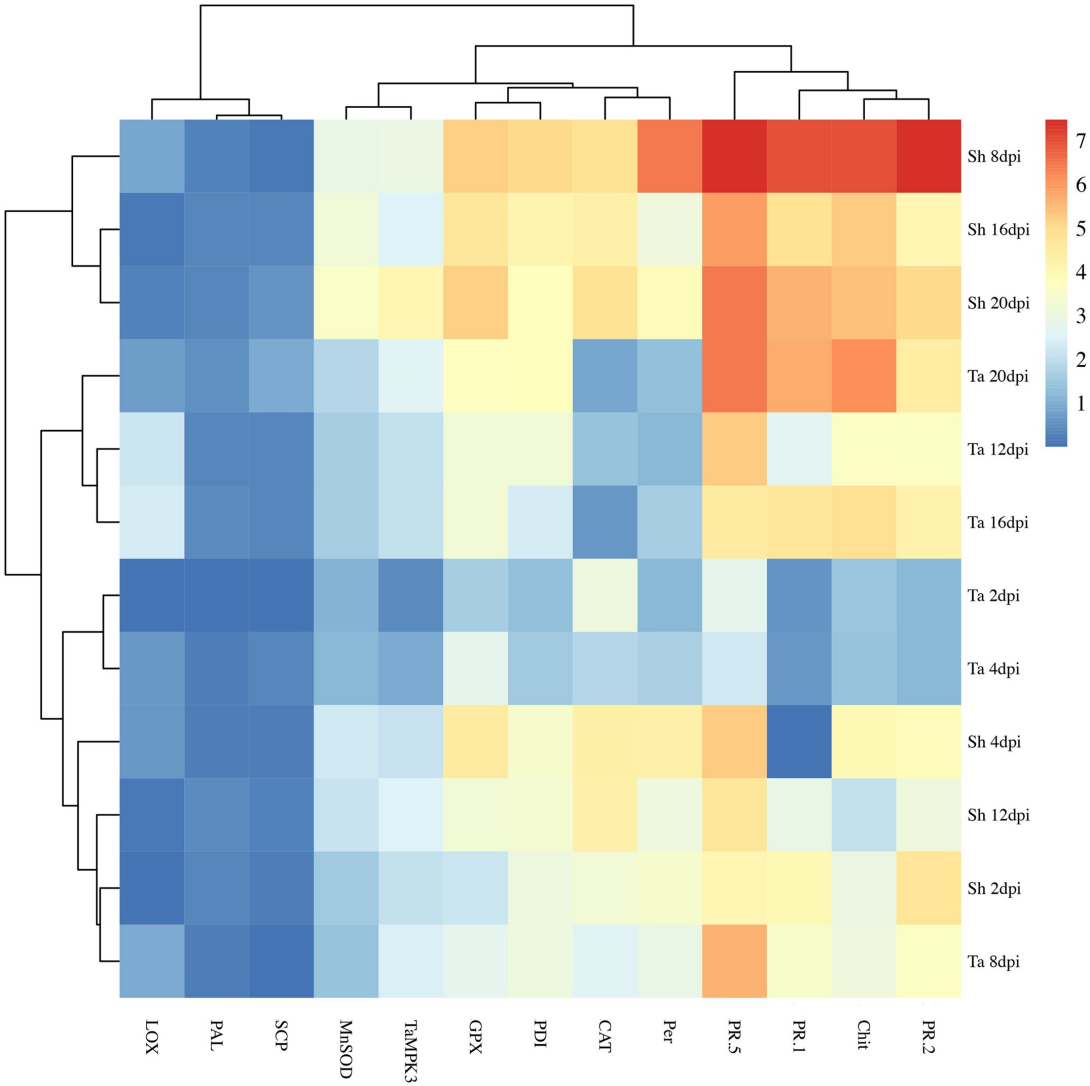
Heatmap analysis signifying expression pattern of selected defense-related genes through R software HTqPCR package to determine readily differential expressed-genes between compatible and incompatible interactions. The R package pheatmap was applied to draw the heatmaps. Number 1 (blue colour) and 7 (red colour) indicate the lowest and highest expression level of tested genes, respectively. Sh represents the cv. Shafir (the resistant cultivar) and Ta indicates the cv. Taichung 29 (the susceptible cultivar). 2, 4, 8, 12, 16 and 21 dpi are selected time courses indicating days post inoculation.

We applied a heat map analysis for better visualization of genes differentially expressed between incompatible and compatible interactions. Our analysis indicated that the expression level of three genes, including *LOX*, *PAL*, and, *SCP* remained without major changes in both interactions while that of five genes (*MnSOD*, *TaMPK3*, *GPX*, *PDI* and *CAT*) had moderate differences in the incompatible and compatible interactions. Interestingly, expression profiles of five genes (*Per*, *PR-1*, *PR-*.*2*, and *PR-5* and *Chit***)** displayed strong differential alternations in the investigated interactions. The highest discriminating expression patterns between the two investigated interactions were observed for *PR-2* and *PR-5* at 8 dpi, corresponding to the switch from the biotrophic phase (Fig 6).

### Targeted quantitative HPLC analysis

Out of 14 targeted polyphenolic compounds, three ones (Table 2; S6 Table) were detected and quantified in both investigated interactions through a targeted quantitative-HPLC technique to obtain novel insights into how these naturally occurring compounds play roles in mediating resistance in the applied wheat cultivars following infection by Z. *tritici* IPO323. Our analysis traced chlorogenic acid, apigenin, and rutin as dominant compounds differentially triggered in the incompatible and compatible interactions. Results are expressed in microgram/gram fresh weight (μg/g fw). Chlorogenic acid was upregulated strongly at 2 and 16 dpi in the susceptible cv. Taichung 29 (compatible interaction) compared with that of resistant cv. Shafir (incompatible context), indicating this compound has a bimodal induction profile. The highest amount of this compound (619 μg/g fw) was observed in the cv. Taichung 29 inoculated by IPO323 at 16 dpi, whereases this compound was low in the incompatible interaction, ranging from 15–121 μg/g fw. Apigenin profiles showed a trimodal pattern, reaching the highest peak at 2, 8 and 21 dpi in the compatible interaction compared with incompatible interaction. The highest amount of apigenin was found to be 185 μg/g fw that was quantified in the Taichung 29 cv. at 8 dpi. Furthermore, this compound was remained almost constant over the tested time courses in the cv. Shafir. Contrastingly, rutin was significantly expressed at 4 and 12 dpi, 66 and 30 μg/g fw, respectively, in the incompatible interaction compared with that of the compatible one, 36 and 19 μg/g fw, respectively. However, rutin was remarkably expressed in the compatible context compared with the incompatible one at other tested time courses and the highest amount of rutin (91 μg/g fw) was traced in the cv. Taichung 29 at 2 dpi (Table 2). This experiment was repeated independently twice to ensure robustness and reliability of our observations.

**Table 2:** Effect of *Zymoseptoria tritici* on (μg/g fw) chlorogenic acid, apigenin, and rutin contents of two contrastingly wheat cultivars that was quantified at various time courses including 2, 4, 8, 12, 16 and 21 days post-inoculation. Data are shown as mean ± SD (Standard Deviation) of three biological replicates.

		Compound name		
Genotype	Time course	Colrogenic acid	Apigenin	Rutin
**Shafir**	2 dpi	15.70k ± 1.62	88.47d ± 2.93	34.16 g±3.78
	4 dpi	40.31j ± 1.90	86.15d ± 2.29	66.95 c±2.50
	8 dpi	74.73fi ± 1.58	74.73f ± 3.58	38.66 f±1.67
	12 dpi	85.37h ± 1.63	86.20d ± 1.32	30.00 h±1.77
	16 dpi	115.94ef ± 16.87	74.82f ± 1.03	35.32 fg±2.05
	20 dpi	121.49e ± 9.90	62.81g ± 2.38	29.93 h±0.88
**Taichung 29**	2 dpi	521.26b ± 17.70	161.15b ± 3.24	90.32 a±1.66
	4 dpi	130.20d ± 9.14	81.79de ± 2.42	36.37 fg±1.71
	8 dpi	108.81fg ± 2.78	185.32a ± 2.21	80.73 b±1.53
	12 dpi	85.45h ± 1.79	47.05h ± 2.15	19.12 i±1.73
	16 dpi	618.73a ± 20.81	72.93f ± 1.92	59.60 d±1.15
	20 dpi	198.51c ± 16.81	131.39c ± 3.42	53.83 e±0.93

## Discussion

In this study, our histopathological study corroborated the previous studies proposing that H_2_O_2_ plays an essential player in *Z*. *tritici*-wheat interaction to restrict and halt the fungal growth in the incompatible interaction and resistant cultivars [35, 41]. It should be noticed that *Z*. *tritici* is a hemi-biotrophic fungus, exploiting two distinct pathogenicity phases to complete its infection cycle [7]. We found that H_2_O_2_ accumulated in the resistant plants to a higher amount at the biotrophic and switching phase in comparison with the susceptible plants (Fig 2A). This observation verified its promising role in halting the fungal growth, similar to that documented previously for the biotrophic fungal pathogens such as *Blumeria graminis* f.sp. *hordei* [36] and for *Z*. *tritici* [35, 41]. Following the transition phase, susceptible plants experienced significant hydrogen peroxide (H_2_O_2_) accumulation in mesophyll cells, coinciding with Septoria tritici blotch (STB) symptom onset and tissue collapse. While H_2_O_2_ typically serves as a signal for resistance, its presence may indicate increased oxidative stress induced by the pathogen. This stress likely exacerbates cellular damage, contributing to STB progression and tissue collapse. Therefore, *Z*. *tritici* may hijack the defense system at the necrotrophic growth stage, as with *Botrytis cinerea* and *Colletotrichum lindemuthianum*, to facilitate the infection process [42, 43].

Here, the genes *Per*, *Chit*, *PR-1*, *PR-2*, and *PR-5* were differentially expressed at 8 dpi, coinciding with the onset of the disease symptom expression and corresponding to the biotrophic switch. *Per* encodes a peroxidase enzyme, playing a central role not only in the diverse physiological processes of plant development but also in plant defense responses through the establishment of a physical barrier or the generation of a highly toxic environment in host tissues to restrict fungal invasion [44] whereas *Chit* encodes a plant chitinase that hydrolyze the N-acetylglucosamine polymer chitin, a key structural component in the cell wall of *Z*. *tritici*. *PR-1* is induced abundantly in host tissues encountered by invading phytopathogenic agents to limit their colonization through its antifungal activity [45] while *PR-2* is a well-known protein to play a pivotal role in plant defense response against biotic stress through degrading of the β-glucan, which is a major polysaccharide in the fungal cell wall and functions as a pathogen associated molecular pattern (PAMP) molecule after release from the cell wall to trigger further defense response [46]. *PR-5* encodes a thaumatin-like protein accumulating to high levels in response to biotic stress and shows antifungal activity in several plant species [47]. *PDI*, encodes a protein disulfide isomerase and catalyzes the generation and breakage of disulfide bonds between cysteine residues within proteins. We anticipated that this gene might play a role in degrading the cysteine-rich proteins secreted by the *Z*. *tritici*, particularly at the transition phase [29].

Our results showed that up-regulation of these five-defense-related genes are strongly induced in the incompatible interaction (Fig 3) suggesting that it might be due to recognition of AvrStb6 secreted by IPO323 through the resistance protein Stb6 present in the cv. Shafir. The transition phase is a turning-point in the battlefield between *Z*. *tritici* and wheat, determining the outcome of interactions in this pathosystem, and these genes could be applied as molecular signatures to define the compatibility/incompatibility in *Z*. *tritici*-wheat relationship. This finding indicates that wheat defense response towards *Z*. *tritici* attack is not solely restricted to the early stage of infection (e.g., the first 24 hours), but rather it is often extended into the transition point when fungal biomass increases dramatically in the compatible interaction [7]. Our results are in disagreement with the study conducted previously by Ray et al., (2003), where *PDI*, *PR-1*, *PR-2* and *PR-5* were differentially upregulated in resistant cv. Tadinia (containing *Stb4* resistance gene) compared with that of the susceptible cv. Yecora Rojo at 12 hours after inoculation (hai) [30]. However, the later time points corresponded with the transition phase and the onset of lesion development was not included in their study. In another study, *PR-1* upregulated strongly in incompatible interaction at early time points, coinciding with the biotrophic stage while it was lowly expressed in the susceptible cultivar [22]. Shetty et al., (2003) indicated that *PR-2* expression was elevated at early time points (1 and 3 dai) in the resistant cv. Stakado while that of was induced in the susceptible cv. Sevin from 9 dai, coinciding with switching phase and entering pathogen to the necrotrophic stage [35]. Interestingly, the highest transcript levels of *Chit* were observed at 9 dpi (transition phase) in the cv. Sevin challenged by IPO323 while it was elevated in the resistant cvs. Tadinia and W7984 inoculated with T48 isolate at early time points. These differences among various studies suggest that the transcript accumulations of the defense-related genes in wheat in response to *Z*. *tritici* attack may depend on combinations of the used host cultivars and fungal genotypes [48].

*CAT* displayed a bimodal expression pattern, reaching a peak at the switching phase (8 dpi) and the late infection phase (21 dpi) in the incompatible interaction (Fig 4). Catalase is one of the most important antioxidant enzymes, playing a vital role in scavenging the ROS molecules such as hydrogen peroxide (H_2_O_2_) accumulated in the infected cells in response to fungal attack. Increasing evidence indicates that oxidative burst, a sudden increase of free radical concentrations in the invaded cells, provide a strong resistance response accompanied with HR reaction against biotrophic plant pathogens to halt further fungal colonization in the attempted sites while an increased ROS level is beneficial for the necrotrophic fungus by triggering HR reaction in such a way to kill more cells [42]. Our result suggested that *CAT* probably plays an essential role in preventing damage to plant cells because the extensive accumulation of H_2_O_2_ generated at 8 and 21 dpi in the incompatible interaction is harmful to plant cells. In addition, results provided in this study revealed that the fine-tune inactivation of H_2_O_2_ is a crucial event in the *Z*. *tritici*-wheat interaction. This event avoids the induction of HR in the biotrophic stage as these free radical functions as a signalling molecule to trigger HR in other pathosystems. Our data is consistent with the previous report demonstrating *CAT’*s transcript remarkably induced in the resistant cv. Stakado infected by IPO323 compared with that of the cv. Sevin [35]. Additionally, gene expression profiling of catalase (*CAT*) in the *Macrophomina phaseolina*-sesame pathosystem revealed dynamic changes in expression levels. Specifically, during the early stage of infection in the incompatible interaction, *CAT* expression was found to be downregulated compared to the compatible interaction. However, at the necrotrophic stage of the incompatible interaction, *CAT* expression showed an upregulation. This differential expression pattern suggested that *CAT* may not be prominently involved in the initial defense response during incompatible interactions but may play a role in managing oxidative stress associated with the necrotrophic phase of the pathogen [49]. It is worth mentioning that the failure of high H_2_O_2_ levels to trigger HR in compatible interactions is likely due to a combination of pathogen-mediated suppression of defense mechanisms, modulation of ROS homeostasis, and co-evolutionary dynamics between plants and pathogens [42, 50].

*GPX*, a key ROS-scavenging enzyme that catalyzes the reduction of H_2_O_2_ to protect the plant cells from oxidative damage generated through oxidative burst events followed the same expression trend similar to that of *CAT*. It was previously demonstrated that the *GPX* expression was significantly upregulated in the moderately resistant cv. Cellule in response to IPO323 at 3 dpi [51]. *SOD* is a critical antioxidant enzyme contributing to scavenge free radicals accumulated in a plant under biotic and abiotic stress conditions, expressed gradually in the resistant cv. Shafir to reach the highest peak at 21 dpi (Fig 4), indicating its potential role in protecting plant cells against oxidative burst events. Keon et al., (2007) monitored the transcript accumulation of *SOD* in the susceptible cv. Riband challenged with the IPO323 and they found that this gene upregulated particularly at the late stage of infection (14 and 21 dpi) [10]. *TaMPK3* was expressed significantly in the incompatible interaction at all investigated time points compared with the compatible interaction (Fig 4). The highest peaks of *TaMPK3* were observed at the transition (8 dpi) and 21 dpi (Fig 4). This result suggested *TaMPK3* probably plays a role in rendering resistance response triggered by Avr-R interaction as stimulation of this gene was reported in the Avr-R-mediated disease resistance reactions [52]. However, these results were inconsistent with the previous report indicating this gene was upregulated remarkedly at the compatible context preceding the onsets of symptom development (8 dpi) [12]. *PAL* was significantly induced in the cv. Shafir at 2 dpi corresponded to the biotrophic stage (Fig 3). This gene encodes the first enzyme implicated in the phenylpropanoid pathway leading to the salicylic acid (SA) biosynthesis [53]. It is worth noticing the plant hormone SA plays an essential role in mediating disease resistance response against the biotrophic phytopathogens. This finding is similar to the previous report demonstrating that *PAL* differentially and significantly triggered in the resistant cv. Tadinia compared with the susceptible cv. Yecora Rojo at the early stage of infection, coinciding with the biotrophic growth [22].

Arrays of molecular players like secondary metabolites are involved in the infected plants to provide an efficient reaction in response to the infection by pathogens. It is well documented that the level of phenolic contents is usually elevated in plants faced with diverse biotic or abiotic stresses [24, 54, 55]. It is well documented phenolic compounds or secondary metabolites are not certainly the only key components of resistance mechanisms in the wheat toward STB. In the current study, we compared the amount of three polyphenolic compounds, including chlorogenic acid, apigenin, and rutin in the resistant and susceptible wheat genotypes in response to *Z*. *tritici* infection (Table 2). Our HPLC analysis revealed that the traced phenolic compounds were elevated markedly in the *Z tritici* infected susceptible background than in the resistant cultivar. In Shafir, the level of phenolic compounds was unchanged without extreme fluctuations during infection except for the apigenin that was significantly increased in the incompatible interaction at 12 dpi compared with that of the compatible one. This result disagrees with the recent study investigating the role of the phenolic compounds in the system complying with the AvrStb6-Stb6 interaction [14]. It seems that the type of plant genotype determines the expression of the phenolic compounds in the *Z*. *tritici*-wheat interaction, as three traced phenolic chemicals here expressed at a high level in the susceptible cv. Taichung 29 compared with the resistant one. The gained data indicates additional players besides other phenolic compounds such as waxes, cuticle thickness, and primary metabolites must contribute to wheat resistance towards STB. Somai-Jemmali et al (2017) measured the total phenolic content of bread wheat (BW) and durum wheat (DW) inoculated by *Z*. *tritici* isolates, and they found no significant differences in phenolic compound content [56]. Furthermore, polyphenol deposition at the attempted infection sites were found on both wheat species. Results provided in this study is consistent with the report on phenolic profiles of raspberry canes in response to infection by *Didymella applanata* and *Leptosphaeria coniothyrium*, where it was shown that the level of phenolic compounds in the canes could be attributed to the differences in disease susceptibility [57]. Additionally, our results are in agreement with reports on the *Venturia inaequalis*-apple interactions, where it was shown that the amounts of phenolic compounds such as apigenin and rutin increased significantly in the susceptible background compared to the control plants [27]. However, there is accumulating evidence displaying the key roles of phenolic compounds in providing resistance towards fungal colonization. It was reported that chlorogenic acid plays an essential role in conferring tomato resistance against *Alternaria alternata*. This response is mediated through inhibiting the alternariol biosynthesis, a central factor to colonize the tomato plants [58]. Furthermore, several studies indicated that rutin and apigenin have an antimicrobial impact on the growth of diverse phytopathogens such as *Aspergillus ochraceus* and *Verticillium dahlia in vitro* [59].

## Conclusion

Ttransition point (8 dpi) is a turning point in mounting compatibility/incompatibility in *Z*. *tritici*-wheat relationship since expression analysis demonstrated that five defense-related genes were remarkably and highly up-regulated in the cv. Shafir at 8 dpi. However, recent studies researching how *Sb6*/*Stb16q* mediated incompatible interaction confirmed that temporal closure of stomata is a key step in hindering the *Z*. *tritici* colonization [14, 15]. We proposed that both events occur in these two crucial stages playing central roles in mediating the incompatible interaction in *Z*. *tritici*-wheat pathosystem. Defense-related genes attributed to the primary metabolites play an important role in providing resistance response against incompatible interaction, while secondary metabolites such as phenolic compounds play an important role in a susceptible genotype to defend themselves, but they are an unsuccessful biological weapon to restrict *Z*. *tritici* colonization in the investigated compatible interaction. However, it seems that the type of applied cultivars defines the type/amount of generated secondary metabolites in this pathosystem based on the results obtained by Seybold et al. 2020, which it was shown that genes implicated in the phenylpropanoids pathway are induced differentially in resistant cultivar (Chinese Spring) at 4 and 8 dpi compared with the susceptible cultivar (Obelisk). To elaborate the presented conclusions in further detail, employing a global transcriptomics/metabolomics approach coupled with a TaqMan real-time PCR to accurately determine the fungal biomass in the inoculated leaves is needed. These would eventually enable us to precisely compare transcripts/metabolites that are differentially accumulated in the incompatible and compatible interactions and to discover instrumental genes mediating wheat resistance towards STB.

## Supporting information

S1 Fig Disease symptoms development on wheat cultivars infected by *Zymoseptoria tritici* IPO323. (a) Plant infected by distilled water (Mock plant). (b) Cultivar Shafir is resistant to IPO323 (incompatible interaction) and no STB symptoms were observed on the inoculated leaves at 21 days post-inoculations. (c) Cultivar Taichung 29 is susceptible to IPO323 (compatible interaction), and necrotic lesions covered by abundant asexual fruiting bodies (pycnidia) were found on the infected leaves at 21 days post-inoculations. The first leaves of the applied cultivars were inoculated with IPO323, and photographs were taken at 21 days post-inoculation.
(TIF)

S1 Table Primers used in this study. (PDF)

S2 Table The targeted phenolic compounds standard used in this study. (PDF)

S3 Table Observed incidence of various developmental phases in the infection course of *Zymoseptoria tritici* IPO323 on cv. Shafir that is resistant to IPO323 (incompatible interaction and cv. Taichung 29 that is susceptible to IPO323 (compatible interaction). Values given are percentages.
(PDF)

S4 Table The raw data generated by PlantCV tool to quantify the DAB and trypan blue staining. (XLSX)

S5 Table The raw data (Ct value) generated during qRT-PCR to profile the expression pattern of the investigated defense-related genes. (XLS)

S6 Table The raw data generated during targeted HPLC to quantify the phenolic compounds identified in the examined interactions. (XLSX)
