# Supplementary figures and images for: Physiological and molecular responses of a resistant and susceptible wheat cultivar to the fungal wheat pathogen Zymoseptoria tritici
Source: PLoS One. 2024 Oct 4;19(10):e0308116. doi: 10.1371/journal.pone.0308116 (PMC11452041; doi:10.1371/journal.pone.0308116)

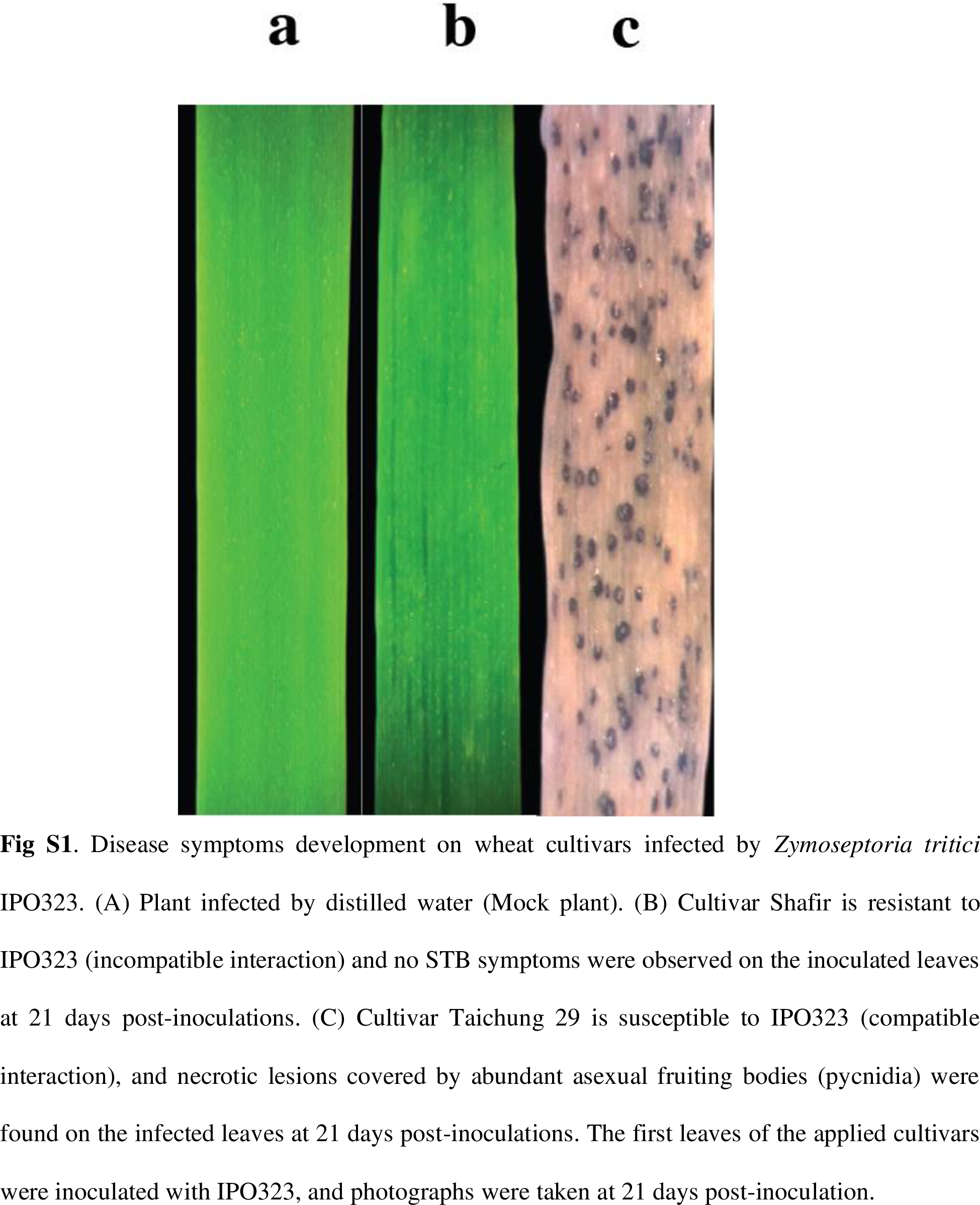

Supplement: S1 Fig — (a) Plant infected by distilled water (Mock plant). (b) Cultivar Shafir is resistant to IPO323 (incompatible interaction) and no STB symptoms were observed on the inoculated leaves at 21 days post-inoculations. (c) Cultivar Taichung 29 is susceptible to IPO323 (compatible interaction), and necrotic lesions covered by abundant asexual fruiting bodies (pycnidia) were found on the infected leaves at 21 days post-inoculations. The first leaves of the applied cultivars were inoculated with IPO323, and photographs were taken at 21 days post-inoculation. (TIF) [file pone.0308116.s001.tif]
