# Supplementary material for: Physiological and molecular responses of a resistant and susceptible wheat cultivar to the fungal wheat pathogen Zymoseptoria tritici
Source: PLoS One. 2024 Oct 4;19(10):e0308116. doi: 10.1371/journal.pone.0308116 (PMC11452041; doi:10.1371/journal.pone.0308116)
Supplement: S1 Table — (PDF) [file pone.0308116.s002.pdf]

Name	Sequence (5'–3') [a]	Reference
PR1-1F	CATGCGATTAGGGACGAAAGA	[28]
PR1-1R	CCGCGGGAATATCATTGG	
PR2-1F	CATGTTCAACGAGAACCAGAAGAC	[28]
PR2-1R	GTATAAGATAGCTACTTGAACTGGATGTTGT	
PR5-1F	GCCGCAAGCCTACCAACA	[28]
PR5-1R	CGCGGTGCGACGTATAGAG	
Per-F	CCAGCACGACACGTGAATG	[20]
Per-R	CATGATTTGCTGCTGCTCGTA	
PAL-F	TGTCCTCTACATTTTTGGTTGCA	[20]
PAL-R	CCTTGACTGCGTTCTTGACATTC	
SCP-F	CCATTACCGATCGCAAATCC	[20]
SCP-R	AGAATCCTATGCTGGGCACTACA	
LOX-F	GGGCACCAAGGAGTACAAGGA	[28]
LOX-R	CGATCACCGACACTCCAATG	
Chit-F	CACACAACTACAACTACG GGCCG	[29]
Chit-R	CGAGCTCTATCGAAACGC CATT	
CAT-F	CCATGAGATCAAGGCCATCT	[30]
CAT-R	ATCTTACATGCTCGGCTTGG	
MnSOD-F	CAGAGGGTGCTGCTTTACAA	[30]
MnSOD-R	GGTCACAAGAGGGTCCTGAT	
GPX-F	CCCCCTGTACAAGTTCCTGA	[30]
GPX-R	GTCAACAACGTGACCCTCCT	
TaMPK3-F	TACATGAGGCACCTGCCG CAGT	[10]
TaMPK3-R	GGTTCAACTCCAGGGCTT CGTTG	
PDI-F	TTATGACTTTGGCCACACCG	[28]
PDI-R	CGAGCTCATCAAATGGCTTG	

S1 Table. Primers used in this study.
