# Supplementary material for: Physiological and molecular responses of a resistant and susceptible wheat cultivar to the fungal wheat pathogen Zymoseptoria tritici
Source: PLoS One. 2024 Oct 4;19(10):e0308116. doi: 10.1371/journal.pone.0308116 (PMC11452041; doi:10.1371/journal.pone.0308116)
Supplement: S2 Table — (PDF) [file pone.0308116.s003.pdf]

No.	Phenolic compound	Migration time (minute)	UV λ max (nm)
**1**	3, 4-dihydroxybenzoic acid	9.3	260
**2**	Catechin	11.3	279
**3**	Chlorogenic acid	14.8	327
**4**	Vanillic acid	16.6	260
**5**	Caffeic acid	16.8	324
**6**	2,5-dihydroxybenzoic acid	17	327
**7**	p-coumaric acid	22.6	310
**8**	Ferulic acid	22.8	323
**9**	Chicoric acid	26.2	330
**10**	Rutin	27	257
**11**	Rosmarinic acid	28.6	330
**12**	Quercetin	34.4	256
**13**	Cinamic acid	34.5	274
**14**	Apigenin	38.6	267

S2 Table. The targeted phenolic compounds standard used in this study.
