# Supplementary material for: Physiological and molecular responses of a resistant and susceptible wheat cultivar to the fungal wheat pathogen Zymoseptoria tritici
Source: PLoS One. 2024 Oct 4;19(10):e0308116. doi: 10.1371/journal.pone.0308116 (PMC11452041; doi:10.1371/journal.pone.0308116)
Supplement: S3 Table — Values given are percentages. (PDF) [file pone.0308116.s004.pdf]

Day 1 Day 2 Day 4 Day 8 Day 12

	**Day 1**			**Day 2**			**Day 4**			**Day 8**			**Day 12**		
	Taichung 29	shafir	Odd Ratio	Taichung 29	shafir	Odd Ratio	Taichung 29	shafir	Odd Ratio	Taichung 29	shafir	Odd Ratio	Taichung 29	shafir	Odd Ratio
Pct germinated Spores	27.40	28.74	0.93^{NS}	20.51	23.55	0.83 ^{NS}	18.74	20.14	0.91 ^{NS}	14.59	17.48	0.81 ^{NS}	16.96	22.22	0.72 ^{NS}
Pct spores with germ tubes growing towards a stoma	4.148	2.66	1.57 ^{NS}	6.29	5.037	1.26 ^{NS}	6.37	6.07	1.05 ^{NS}	8	7.04	1.15 ^{NS}	6.44	5.62	1.15 ^{NS}
Pct spores with germ tubes ending on a stoma	1.11	1.48	0.75 ^{NS}	4	3.11	1.29 ^{NS}	5.41	4.81	1.13 ^{NS}	6.44	5.55	1.17 ^{NS}	5.03	4	1.27 ^{NS}
Pct spores with germ tubes growing past a stoma	0.66	0.51	1.28 ^{NS}	2.07	1.629	1.27 ^{NS}	2.81	2.29	1.23 ^{NS}	4.29	3.25	1.33 ^{NS}	4.88	2.22	2.26 ^{NS}

S3 Table. Observed incidence of various developmental phases in the infection course of *Zymoseptoria tritici* IPO323 on cv. Shafir that is resistant to IPO323 (incompatible interaction and cv. Taichung 29 that is susceptible to IPO323 (compatible interaction). Values given are percentages.
